# Nonengraftment donor lymphocyte infusions for refractory acute myeloid leukemia

**DOI:** 10.1038/bcj.2015.100

**Published:** 2015-12-04

**Authors:** J L Reagan, L D Fast, M Nevola, K Mantripragada, A Mulder, F H J Claas, K Rosati, A Schumacher, H Safran, C T Young, M I Quesenberry, E S Winer, J N Butera, P J Quesenberry

**Affiliations:** 1 Division of Hematology and Oncology, Department of Medicine, Rhode Island Hospital, The Warren Alpert Medical School of Brown University, Providence, RI, USA; 2 Department of Immunohematology and Blood Transfusion, Leiden University Medical Center, Leiden, The Netherlands; 3 Brown University Oncology Group, The Warren Alpert Medical School of Brown University, Providence, RI, USA; 4 Lifespan Office of Clinical Research, Rhode Island Hospital/The Miriam Hospital, Providence, RI, USA; 5 Rhode Island Blood Center, The Warren Alpert Medical School of Brown University, Department of Pathology, Providence, USA

Allogeneic stem cell transplantation (allo-SCT) is an effective treatment modality for acute myeloid leukemia (AML) that exerts its therapeutic benefit via a graft versus leukemia effect.^1^ Its applicability is limited by toxicity from preconditioning chemoradiation in addition to the threat of graft versus host disease, which precludes many patients with AML from receiving this definitive consolidative therapy.^2^ Previously, we have shown an ability to generate responses, some of which were complete, in patients with relapsed/refractory AML using haploidentical donor lymphocyte infusions in the absence of engraftment.^3^ We present data from our FDA-approved Phase II clinical trial (BrUOG H-273, IND 015008, NCT01685606) in which haploidentical allogeneic cells are infused. Unlike our previous trial these cellular infusions occur without granulocyte colony stimulating factor (G-CSF) mobilization or preconditioning low-dose total body irradiation (TBI). The goal is to generate a robust allogeneic response that breaks host tumor tolerance without the toxicity profile seen with allo-SCT.

This trial was approved by the Rhode Island Hospital Institutional Review Board. Eligible patients had relapsed or refractory AML without curative options. Family members who were human leukocyte antigen (HLA) haploidentical donors at the A/B/DR loci were identified. Both patients and donors were consented. Donors then underwent leukapheresis without stem cell mobilization. In all, 1–2 × 10^8^ CD3+ cells/kg were infused unprocessed immediately following collection. Peripheral blood samples were collected on days 2, 7 and 14 post-cellular infusion for short tandem repeat chimerism analysis. Additional peripheral blood samples were collected pre-infusion as well as at 0–4, 8–24, 34–48, 72–96 and 168–192 h post infusion to evaluate recipient effector cells and cytokine release profiles. Following centrifugation to obtain plasma, peripheral blood mononuclear cell (PBMNC) were obtained using Ficoll–Hypaque discontinuous centrifugation. Recipient versus donor cells in the PBMNC were identified by the use of donor-specific anti-HLA antibodies that had previously been used to detect microchimerism during pregnancy.^4, 5^ Recipient PBMNC T-cell subpopulations were characterized for activation markers, cytolytic markers and checkpoint inhibitors. In addition, CD273 (PD-L2) and CD274 (PD-L1) expression on leukemic blasts was determined. Cytokine (IL-2, IL-6, IL-10, IFNγ) levels present in the plasma were determined using the CD8 Milliplex assay (EMD Millipore, Darmstadt, Germany). Wilcoxon rank-sum test was used to determine statistical significance using STATA /SE 12.1 (StataCorp LP, College Station, TX, USA).

Five patients (age 52–77 years, 1–4 previous therapies) were infused with haploidentical donor cells. Four developed hyperpyrexia post infusion that lasted 24–48 h (median temperature maximum (*T*_max_) 0–4 h post infusion 98.6 °F, median *T*_max_ 8–192 h post infusion 101.9 °F, *P*=0.009). Recipient CD8 T cells demonstrated decreased perforin expression post infusion compared with pre-infusion with no changes in granzyme A/B, LAMP-1 or FasL expression. Recipient CD4 T cells showed increased LAMP-1 expression post infusion compared with pre-infusion with no changes in perforin, granzyme A/B or FasL expression. Rapid upregulation of PD-1 on host CD8 T cells was present (Table 1). The greatest difference in PD-1 expression was pre-infusion compared with 60–96 h post infusion. Non-statistically significant upregulation of surface PD-1 ligands occurred on CD33+ leukemic blasts from 0–4 (median 6.9%) to 8–24 h post infusion (median 26.9%) for PD-L1, and 0–4 (median 11%) and 72–96 h post infusion (median 28.6%) for PD-L2. No change was seen in PD-1 expression of recipient CD4 T cells. Likewise, no changes in CTLA-4, OX40, 4–1BB and CD40L expression on recipient CD8 and CD4 T cells pre- or post infusion were observed.

A statistically significant increase in IFNγ immediately post infusion and IL-2 at the onset of fever development (8–24 h post infusion) was seen (Table 2). Two patients developed grade 4 neutropenia whereas one patient developed grade 4 lymphocytosis. There were no signs of graft versus host disease. No dose-limiting toxicities or durable chimerism was seen as all patients had <2% chimerism by day 7. One of the five patients demonstrated a decrease in marrow blast counts post therapy (43% pre- to 21% 8 weeks post infusion).

Overall survival post donor lymphocyte infusion ranged from 21.5 to 46 weeks. One patient was excluded from survival analysis as the patient died before a protocol amendment to collect survival data. One patient is still alive with AML at 46 weeks post infusion. This patient had stable disease following cellular infusion.

Haploidentical cellular infusions are well tolerated and demonstrate biological activity in relapsed AML. Notably, we saw less fever development as well as a decrease in clinical responses with this clinical trial compared with the prior trial. Herein, 1/5 patients had a transient response whereas previously 10/13 patients with AML had a transient response.

Laboratory correlative studies performed during the trial offer some insight into why a decrease in response was witnessed and provide a rationale by which leukemic blasts evade the host immune system. In this study, cytokine release profiles post infusion were consistent with an immune inhibitory profile with high IL-10 expression and low IFNγ, IL-2 and IL-6 levels. In our previous study, post-infusion median IL-10 levels were similar, however, the inflammatory cytokines IFNγ and IL-6 were much more pronounced (median 30.9 pg/ml and median 3757 pg/ml). We therefore hypothesize that fever development with resultant cytokine release, especially IL-6, at least correlates with anti-leukemia response. Given the paucity of responses seen here, we conclude that either donor G-CSF mobilization, TBI or both are necessary for fever and subsequent cytokine release. Whether or not the fever and IL-6 directly cause response remains to be seen.

Interestingly, lab correlative studies provided insight into recipient T-cell interactions with leukemic blasts as a potential rationale for lack of response. In the present study, non-mobilized donor lymphocyte infusions initially generated a fever response and degranulation of recipient T cells as evident by the decrease in perforin expression pre- compared to post-cellular infusion. Any significant leukemia cell killing appears to be silenced through upregulation of PD-1 on recipient T cells and PD-1 ligands on leukemic blasts. Upregulation of the PD-1/PD-L1 axis has been previously shown as a mechanism of immune escape^6^ with PD-1 cytotoxic T-cell suppression further enhanced through regulatory T cells in murine models of AML.^7^ Furthermore, increase in minor histocompatibility-specific memory CD8 T-cell expression of PD-1 with simultaneous increase in PD-L1 leukemic blast production has been described as a mechanism of immune evasion in allo-SCT patients who relapse^8^ and that IFNγ production can upregulate PD-L1 expression on leukemic blasts in AML patient samples.^8, 9^ Here, in addition to increased PD-L1 we also show an increase, albeit not statistically significant, in PD-L2 within leukemic blasts. Although the upregulation of PD-L2 on dendritic cells (DCs)^10^ and the ability of leukemic blasts to differentiate into DCs^11^ have both been well described, the presence of PD-L2 on leukemic blasts has, to our knowledge, not been reported. The co-expression of PD-1 ligands at different time points may reflect an initial upregulation of PD-L1 followed by a later increase in PD-L2 reflective of the relative increase in IFNγ thereby underscoring the complex immune evasive ability of AML.

In summary, haploidentical lymphocyte infusions provide a strong initial immune stimulus. It is unclear why there was no leukemia control in the absence of G-CSF mobilization and TBI. Conclusions are limited as the earlier protocol with G-CSF and TBI did not examine changes in the PD1/PDL1/2 axis. Much further work is needed to identify the mechanisms and develop what may be a promising low-intensity treatment strategy for AML.

## Figures and Tables

**Table 1:** Median recipient T-cell activation, cytolytic and immune checkpoint markers pre- and post-haploidentical donor lymphocyte infusion

	*Pre-infusion*	*Post-infusion*	P*-value*
*CD8*			
Perforin	77.6	61	***0.03***
Granzyme A	78.1	73.6	0.15
Granzyme B	60.8	66.3	0.90
LAMP-1	15.6	32.7	0.13
FasL	9.5	14.3	0.34
PD-1	5.6	49.7	***0.005***
			
*CD4*			
Perforin	13.0	5.3	0.23
Granzyme A	74.5	63.1	0.41
Granzyme B	3.7	10.1	0.41
LAMP-1	3.3	8.6	***0.03***
FasL	13.6	6.3	0.10
PD-1	13.0	18.4	0.41

Values that are significantly different are in bold and italics.

**Table 2:** Median pre versus post cytokine release profiles following haploidentical donor lymphocyte infusion

*Time from infusion*	*IFNγ*	P-*value*	*IL-2*	P-*value*	*IL-6*	P-*value*	*IL-10*	P-*value*
Pre-infusion	0.001		4.5		12.65		81.25	
*Post infusion*								
0–4 h	0.001	0.37	2.83	0.10	4.66	0.81	73	0.62
8–24 h	7	**0.03**	56.3	**0.02**	25.8	0.46	171	0.8
34–48 h	10.25	0.13	34.93	0.08	22.17	0.15	172.45	0.25
72–96 h	6.4	0.13	5.4	0.46	12.59	1.00	259.9	0.22
168–192 h	1.0	0.13	1.35	**0.05**	9.5	1.00	170.6	0.56

Bold numbers are statistically significant.
